# On the Treatment of Asphyxia Neonatorum

**Published:** 1849-09

**Authors:** 


*On the Treatment of Asphyxia Neonatorum.*—By J. O. FLETCHER, Esq., Manchester.

[Refering to the plan of treating still-born children by the use of warm and cold water alternately, Fletcher, says:]

I have been in the habit for some years of treating all such cases in a very similar way, and with great success. I first immerse the child in warm water, and, upon withdrawing it, cover the chest with a cloth or sponge well soaked with cold water (the colder the better:) again immerse it in warm water, and again apply the cold water, so on alternately using the hot and cold water, until there is evidence of respiratory movements. The first applicatjon of cold will generally produce a slight sob, and repeated applications will establish respiration I conceive the good arises from the sudden impression caused by the cold on the cutaneous nerves, (which are the principal) "*excitor nerves*" in the reflex action of respiration. This is followed by response along the "*motor nerves*" of this function as the phrenic, intercostal &c.; hence the sob on the first application, and the establishing of respiration by being repeated. I have for an equally long period, been in the habit of ligating the cord before the complete birth of the child, in breech and feet presentations, sometimes even before the pulsations were obliterated, believing, as I do, that the child in these cases dies from hemorrhage into the placenta, arising from the umbilical vein being much exposed to pressure, by virtue of its superficial and unprotected positionin the cord, which, together with the tenuity of its tunics render it very liable to have its current obliterated, whereas the tunics of the umbilical arteries are firmer, and they themselves much exposed; thus they are in a measure protected from the consequences of slight pressure. Therefore, the flow of arterial blood through the vein may become obliterated, whilst the venous blood continues to flow along the arteries, from the child, into the placenta, without there bring any counterbalancing stream; hence the great mortality in these cases by the usual treatment, and hence the utility of ligating the cord early, thereby removing one fatal consequence; and, as it is well known that a child can breathe in the vagina, its chances of life are not to say the least dimimished, but, I think, much increased; for out of thirty-seven cases that I have treated in this way, *two children only have died*, which is saying very much more than I can say for the usual treatment. In this class of cases especially, I think the good effects of alternate application of cold and warm water will be seen, if tried.—*Medical Times.*

